# Weight gain with olanzapine: Drug, gender or age?

**DOI:** 10.4103/0019-5545.31617

**Published:** 2006

**Authors:** Sanjay Jain, Manish Bhargava, Shiv Gautam

**Affiliations:** * Associate Professor Psychiatric Centre, Department of Psychiatry, S.M.S. Medical College, Jaipur, Rajasthan; ** Resident Psychiatric Centre, Department of Psychiatry, S.M.S. Medical College, Jaipur, Rajasthan; *** Professor and Head Psychiatric Centre, Department of Psychiatry, S.M.S. Medical College, Jaipur, Rajasthan

**Keywords:** Weight gain, olanzapine, body mass index, age, gender, dose

## Abstract

**Background:**

The introduction of atypical antipsychotics was a big step forward in the treatment of schizophrenia and other psychoses. Their limitations, however, became evident over time.

**Aim:**

To study the causes of weight gain associated with the use of olanzapine—an atypical antipsychotic drug.

**Methods:**

Eighty patients fulfilling the ICD-10 criteria for schizophrenia, predominantly with negative symptoms, were included in this study to evaluate weight gain as an adverse effect of treatment with olanzapine in relation to age, gender, dose and body mass index (BMI). Sociodemographic data and baseline weight along with height (to calculate the BMI) were recorded before the initiation of treatment. The patients were administered a flexible dose of olanzapine (5—15 mg) as monotherapy. Pregnant patients, smokers and those with endocrine disorders, cardiac problems and organic brain dysfunction were excluded from the study. The increase in weight as a neuroleptic side-effect of olanzapine was recorded and analysed in relation to age, gender, dose and BMI.

**Results:**

Of the patients receiving olanzapine, 66.6% had a weight gain of 1–5 kg over a period of 4 weeks. The weight gain was not related to the dose of the drug or BMI. The interesting finding was that the increase in weight was significantly related to age ≥40 years and female sex, indicating that women ≥40 years of age are more prone to gain weight with olanzapine therapy in comparison with women <40 years and men of any age group.

**Conclusion:**

The potential for weight gain associated with the use of atypical antipsychotics to cause long-term complications will need further study. Clinicians are encouraged to monitor weight, plasma glucose and leptin levels, and lipid parameters in patients receiving olanzapine.

## INTRODUCTION

The introduction of antipsychotic drugs during the 1950s was a big step forward in the treatment of schizophrenia and other psychoses of these drugs effectiveness as well as adverse effect profile, however, became evident over time. Until a few years ago, these novel antipsychotics demonstrated an improved therapeutic profile compared with that of conventional antipsychotics in terms of effectiveness and adverse effect profile. With regard to endocrine and metabolic effects, there are no surprises at this stage with conventional antipsychotics. The adverse effect profile of these drugs is well known and can generally be anticipated. Accordingly, clinicians are now focusing away from concerns of motor effects and their emphasis has shifted to weight gain, drug-induced glucose imbalance and other emerging metabolic effects associated with the use of atypical antipsychotics.

A national survey in the USA, which evaluated the patterns of use and emerging adverse effect profile of atypical antipsychotics, found that 70% of patients were prescribed an atypical antipsychotic drug and only 30% a typical antipsychotic. Thirty-four per cent of the patients on atypical antipsychotics reported weight gain in comparison to 16% on typical antipsychotics; weight gain was more evident in females (54%).^1^

Since weight gain contributes to non-compliance with treatment and may lead to medical morbidity, it is important to know and study the causes of weight gain associated with the use of the atypical antipsychotic olanzapine.

Olanzapine, a thienobenzodiazepine, is an atypical antipsychotic drug with a high affinity for the serotoninergic receptors 5-HT_2_ and 5-HT_6_, and a low affinity for 5-HT_3_ receptors *in vitro.* It also has a high affinity for dopaminergic receptors, mainly D2, D3 and D4; muscarinic M1–5; α_1_ adrenergic; and histaminergic H1 receptors *in vitro.*^2^ The drug reaches peak plasma levels in 5–8 hours and is metabolized through cytochrome p450 cyp1a2 and p450 cyp2d6. It has a half-life of about 45 hours, depending on the rate of metabolism. The recommended dosage is 20 mg daily, but higher doses have been used. The most common side-effects are somnolence and weight gain.

## METHODS

The study was conducted at the Psychiatric Centre of SMS Medical College, Jaipur. Eighty consecutive outpatients who presented at the Department of Psychiatry from April to November 2001 and who were diagnosed as having schizophrenia or its subtypes according to the ICD-10 criteria, either fresh or clinically non-responders to typical or other atypical antipsychotics, were included in the study. Patients with co-morbid medical illness related to the heart, liver, thyroid, kidney, brain, pancreas; pregnant patients; and smokers were excluded from the study. Informed consent was taken from the patients or their legal caretakers before initiation of the study. Previously given antipsychotics, if any, were slowly titrated and in the drug-free period of 15 days, they were given only benzodiazepines. A complete baseline investigation was done; clinical symptoms were examined; and the weight and body mass index (BMI) were recorded in a predesigned proforma. The patients were started on olanzapine monotherapy (in doses of 5–15 mg; mean dose 8.375 mg); only benzodiazepines were used as concomitant medication. The patients' weight was recorded bi-weekly till the end of the fourth week. The change in weight was recorded and data on weight gain with respect to age, gender, BMI and dose were analysed.

## RESULTS

Of the 80 subjects, only 60 (30 men and 30 women) completed the study. The median age of the patients was 39.3 years (age range: 18–48 years). The patients were divided in two groups according to age (Table 1). There were 30 patients in each group; group I had 16 men (53.3%) and 14 women (46.6%) <40 years of age, and group II had 14 men (46.6%) and 16 women (53.3%) ≥40 years of age.

**Table 1:** Distribution of the natients according to age and gender (*n*=60)

	Age group			
			
Gender	<40 years	≥40 years	Total patients
Mies	16 (26.6)	14 (23.3)	30 (50)
Females	14 (23.3)	16 (26.3)	30 (50)
Total	30	30	60

Values in parentheses are percentages.

### Weight change

Of the 60 patients who completed the study, 40 (66.6%) gained weight; 18 patients (30%) (mean weight gain: 2.3±2.7 kg) were <40 years of age, whereas 22 (36.6%) (mean weight gain: 3.5±2.11 kg) were ≥40 years. The two groups were compared and the results were statistically significant at p<0.01, indicating that patients ≥40 years of age are more prone to gain weight (Table 2).

**Table 2:** Distribution and comparison of weight gain according to age (*n*=60)

Age group (years)	patients who gained weight (*n*)	No weight gain (*n*)	Total	Mean weight gain±SD (kg)
<40	18 (30)	12 (20)	30	2.3±2.7
≥40	22 (36.6)	8 (13.3)	30	3.5±2.11
Total	40 (66.6)	20 (33.3)	60	

Values in parentheses are percentages, *t* test (tail 2; type 3) = 2.08; p<0.05

Among women, 24 (80%) gained weight; 9 (30%) were <40 years of age (mean weight gain: 1.52±1.41 kg) and 15 (50%) were ≥40 years (mean weight gain: 2.75±1.29 kg). When the two groups were compared using the *t* test, the results were statistically significant (p<0.01), indicating that women ≥40 years of age are more prone to gain weight (Table 3). Among men, 16 (53.3%) gained weight; 9 (30%) were <40 years of age (mean weight gain: 0.87±0.95 kg) and 7 (23.3%) were <40 years of age (mean weight gain: 0.71±0.69 kg). The two groups were compared using the *t* test; the results were not statistically significant (p>0.1), indicating that men are prone to gain weight irrespective of the age (Table 4). When change in weight was compared on the basis of gender, only 40% of men gained weight in comparison with 60% of women, which was statistically significant (p<0.01), indicating that women patients are more prone to gain weight in comparison with men (Table 5).

**Table 3:** Distribution and comparison of women according to weight gain and age (*n*=30)

Age group (years)	Patients who gained weight (*n*)	No weight gain (*n*)	Total (*n*)	Mean weight gain±SD (kg)
<40	9(30)	5(16.6)	14(46.6)	1.52±1.41
≥40	15 (50)	1 (3.3)	16 (53.3)	2.75±1.29
Total	24(80)	6(20)	30(100)	

Values in parentheses are percentages, *t* test (tail 2; type 3) = 2.40; p<0.05

**Table 4:** Distribution and comparison of men according to weight gain and age (*n*=30)

Age group (years)	Patients who gained weight (*n*)	No weight gain (*n*)	Total (*n*)	Mean weight gain±SD (kg)
<40	9 (30)	7 (23.3)	16 (53.3)	0.87±0.95
≥40	7 (23.3)	7 (23.3)	14 (46.6)	0.71±0.82
Total	16 (53.3)	14 (46.6)	30 (100)	

Values in parentheses are percentages, *t* test (tail 2; type 3) = 0.44; p<0.1

**Table 5:** Comparison of weight gain in patients accoiding to gender (*n*=60)

Gender	Patients who gained weight (*n*)	No weight gain (*n*)	Total (*n*)	Mean weight gain±SD (kg)
Miles	16 (26.6)	14 (23.3)	30 (50)	0.77±0.91
Females	24 (40)	6(10)	30 (50)	2.13±1.2
Total	40 (66.6)	20 (33.3)	60(100)	

Values in parentheses are percentages, *t* test (tail 2; type 3) = 2.72; p<0.01

### Weight gain in relation to dosage

The distribution of dosage in the 60 patients is shown in Table 6. The mean weight gain in each group is also listed in Table 6. The groups were compared with each other using the *t* test (tail 2, type 3). The *t* values were 0.89 (5–10 mg), 0.76 (10–15 mg) and 0.84 (5–15 mg), which were not statistically significant.

**Table 6:** Distribution of weight gain accoiding to the dosage (*n*=60)

Dosage (mg/day)	No weight gain (*n*)	Weight gained (*n*)	Total (*n*)	Mean weight gain±SD (kg)
5±2.5	10 (16.6)	16 (26.6)	26 (43.3)	1.47±1.6
10±2.5	5 (8.3)	17 (28.3)	22 (36.6)	1.4±1.1
15±2.5	5 (8.3)	7(11.6)	12 (20)	1.5±1.2
Total	20 (33.2)	40 (66.32)	60 (100)	

Values in parentheses are percentages

### Weight gain in relation to BMI

The BMI increased in 40 patients (66.66%). Twenty-four were women (40%); 5 (8.33%) had a BMI below the recommended level (<25). Among men, 16 (26.6%) had an increase in the BMI, but only 1 (1.6%) had a BMI below the recommended level (<27). The mean change in the BMI was 0.25 in men and 0.89 in women (Table 7). There was no statistically significant value, suggesting that patients with a low BMI are more prone to gain weight.

**Table 7:** Comparison of weight gain in patients accoiding to the body mass index (BMI) (*n*=60)

Gender	Patients who gained weight (kg) (*n*=40)	Mean BMI (pretreatment)	Mean BMI (pretreatment)	Mean BMI (gain)
Mies	16 (26.6%)	27.12	27.37	0.25	
Females	24 (40%)	25.39	26.28	0.89

## DISCUSSION

The findings were similar to those of various studies which have also reported weight gain with olanzapine.^3^–^7^ The principal findings of this study were that 66.6% of the patients exhibited a change in weight; the majority were ≥40 years of age. There was a significant difference between the two sexes; women ≥40 years of age were more prone to gain weight in comparison with those <40 years and men of any age group. This is contrary to the finding of the study by Melkersson *et al.*^3^ They reported weight gain and increase in BMI during olanzapine treatment but did not find any difference between the two sexes. Another report which was found to be contrary was by Wetterling *et al.,*^8^ who said that ‘the young and not obese show the highest weight gain.’ The finding that women have a greater tendency to gain weight was similar to that reported by Gopalaswamy *et al.*^9^ But Kinon *et al.*^10^ reported that men are more prone to gain weight. In relation to the BMI, there was no significant change in weight in patients having a low BMI at baseline, which was in contrast to the findings of Tollefson *et al.*^11^ They observed that olanzapine-related weight gain was more common among patients with a low baseline BMI. In relation to the dose-dependent weight gain, there was weight gain with a flexible dose of olanzapine. This was similar to the findings of Conley *et al.*^12^ but contradictory to those reported by Nemeroff *et al.*^4^ and Green,^13^ in which he states that about 40% of patients gain weight if they are on a high starting dose and if they are underweight before treatment. An interesting finding of our study was that there was no significant relationship between the BMI and weight gain.

A review of the literature was done to find out the possible mechanisms of weight gain with olanzapine. These are listed in Box 1.

**Box 1:** Possible mechanisms behind weight gain with olanzapine

Improved functioning-Jess self-neglect, improved eating habits Increase in appetite-eaibohydrate craving, sedation and decreased motor activity Altered adipose tissue-Hetabolism, fat composition (brown/white/adipose tissue) and distribution Neurotransnitter effects due to serotonineigic, histamineigic,^14^ dopanineigic, adreneigic (alpha- 1) receptor blockade or a combination of these^6^,^7^ Increase in the leptin level^15^,^16^ Hyperinsulinaemia, hyperlipidaemia, insulin resistance and hyperleptinaemia may be associated with weight gain^3^ Increase in prolactin level^17^

The association of olanzapine with weight gain and the potential for long-term complications will need further study so that adequate measures can be initiated to prevent significant morbidity and mortality as patients with schizo-phrenia have a tendency to gain weight and acquire related medical disorders. Also, systemic research with more rigour is needed to gather information on the increased susceptibility of women patients >40 years of age to gain weight.

### Limitations

The initial findings of an ongoing study have been reported here. The main limitation of this study is that a control group comprising women matched for age and treated with placebo/other antipsychotics was not used and neither was a periodic hormonal assay done to establish the reasons for weight gain with olanzapine. Also, the duration of the study was short.

## CONCLUSIONS

The results of the study revealed that there is a significant gain in weight with olanzapine, which is related to age ≥40 years and sex; women ≥40 years of age are more prone to gain weight in comparison with those <40 years and men of any age group. The weight gain is not related to the dose of olanzapine and BMI. Of the 40 patients who gained weight, 23 (57.5%) reported an increase in appetite. The association of atypical antipsychotics with weight gain and their potential to cause long-term complications will need further study to prevent and diagnose these complications early so that adequate measures can be initiated. Clinicians are encouraged to monitor weight, plasma glucose and leptin levels, and lipid parameters in patients receiving olanzapine.
